# Direct observation of local xylem embolisms induced by soil drying in intact *Zea mays* leaves

**DOI:** 10.1093/jxb/erw087

**Published:** 2016-03-05

**Authors:** Jeongeun Ryu, Bae Geun Hwang, Yangmin X. Kim, Sang Joon Lee

**Affiliations:** ^1^Department of Mechanical Engineering, Pohang University of Science and Technology (POSTECH), Pohang, Gyeongbuk, 790-784, Republic of Korea; ^2^Center for Biofluid and Biomimic Research, Pohang University of Science and Technology (POSTECH), Pohang, Gyeongbuk, 790-784, Republic of Korea

**Keywords:** Drought stress, embolism, soil drying, xylem, X-ray imaging, *Zea mays* (maize).

## Abstract

The vulnerability of vascular plants to xylem embolism is closely related to their stable long-distance water transport, growth, and survival. Direct measurements of xylem embolism are required to understand what causes embolism and what strategies plants employ against it. In this study, synchrotron X-ray microscopy was used to non-destructively investigate both the anatomical structures of xylem vessels and embolism occurrence in the leaves of intact *Zea mays* (maize) plants. Xylem embolism was induced by water stress at various soil drying periods and soil water contents. X-ray images of dehydrated maize leaves showed that the ratio of gas-filled vessels to all xylem vessels increased with decreased soil water content and reached approximately 30% under severe water stress. Embolism occurred in some but not all vessels. Embolism in maize leaves was not strongly correlated with xylem diameter but was more likely to occur in the peripheral veins. The rate of embolism formation in metaxylem vessels was higher than in protoxylem vessels. This work has demonstrated that xylem embolism remains low in maize leaves under water stress and that there xylem has characteristic spatial traits of vulnerability to embolism.

## Highlight

Direct X-ray micro-imaging reveals localized xylem embolism under water stress conditions in the leaves of intact maize, with a specific spatial distribution of embolism in terms of xylem structure.

## Introduction

Vascular plants draw sap water from the soil to their leaves using negative pressures created by leaf transpiration (Dixon and Joly, 1894). However, negative hydrostatic pressure has adverse effects on water transport in xylem vessels, causing the water to be in a metastable state and become prone to embolism (Tyree and Sperry, 1989; Holbrook *et al.*, 2002; Stroock *et al.*, 2014). Embolism will also block water transport in xylem vessels when plants experience drought, freezing–thawing stresses, or insect-borne damage. Current concerns about the effects of global warming (Choat *et al.*, 2012) have aroused great interest in embolism occurrence and drought risk in plants. Although vascular plants have vulnerable transport systems, they are known to have drought avoidance, tolerance, and escape strategies to cope with water-limited environments (Vilagrosa *et al.*, 2012).

To date, a number of studies have measured xylem cavitation and embolisms, employing acoustic emissions from cavitation (Milburn and Johnson, 1966), observations of xylem water content in thin wood sections (Sperry, 1985; Canny, 1997), and indirect hydraulic measurements (Sperry *et al.*, 1988). However, recent studies (Cochard *et al.*, 2013; Wheeler *et al.*, 2013; Rockwell *et al.*, 2014) have reported that existing measurement techniques on xylem embolism and vulnerability to cavitation may be prone to artefacts. Xylem embolism can be indirectly evaluated by measuring hydraulic conductance loss, but this measurement can cause air entrainment in xylem vessels during sampling. This process also disrupts the vascular networks of intact plants, which may overestimate embolism occurrence in xylem vessels. Ultrasonic acoustic detection also creates artefacts originating from the obscurity of the origin of acoustic emission signals (Jackson and Grace, 1996). Similar to these methods, the use of cryo-SEM for detecting xylem embolism has been criticized because freezing tissues may disturb the natural situation (Cochard *et al.*, 2000; Johnson *et al.*, 2009). To overcome these technical limitations, imaging techniques such as MRI and X-ray imaging have recently been used in studies of xylem embolism and the structural traits of xylem vessels associated with embolism in intact plants (Scheenen *et al.*, 2007; Kaufmann *et al.*, 2009; Brodersen *et al.*, 2010; Brodersen *et al.*, 2013a; Brodersen *et al.*, 2013b; Choat *et al.*, 2015; Cochard *et al.*, 2015). Direct measurements of xylem embolism in recent studies showed that it is possible to further study the water status of intact monocot plants using X-ray imaging techniques.

In this study, we aimed to non-destructively monitor the water content of xylem vessels in leaves of intact maize (*Zea mays.* L) under systematic soil drying using a synchrotron X-ray micro-imaging technique. This X-ray imaging technique (Lee and Kim, 2008; Brodersen *et al.*, 2010) enabled us to investigate the water content in individual xylem vessels with a spatial resolution of around 1 μm, compared with studies on sap flow and embolism in xylem vessels using MRI, which has a typical resolution of around 50 μm (Holbrook *et al.*, 2001; Köckenberger, 2001; Zwieniecki *et al.*, 2013). In addition, by inducing soil-drying treatments on intact sample plants, we were able to surpass the current level of examination of excised leaves or roots using the X-ray micro-imaging technique (Lee and Kim, 2008; Kim and Lee, 2010; Lee *et al.*, 2013; Yanling *et al.*, 2013; Ryu *et al.*, 2014). Based on consecutively captured X-ray images, we directly observed embolisms in xylem vessels at a high spatial resolution, and quantitatively analysed incidence rates of embolism occurrence in terms of the anatomical structure of xylem vessels, such as diameter, type, and vessel position in leaves. Characteristic spatial distributions of xylem embolism revealed in this study provide some clues to the drought-tolerance mechanism, demonstrating that there is strong resistance to xylem embolism in plants. Direct observations of the biophysical phenomena also excluded possible artefacts encountered in indirect measurements of xylem embolism, and facilitated the examination of drought-induced embolism in xylem vessels of intact crop plants.

## Materials and methods

### Plant materials and growth conditions

Maize (*Zea mays* L.) was chosen for this study because it is a crop plant in which cavitation is thought to occur under moderate water stress (Tyree *et al.*, 1986; McCully *et al.*, 1998). Three- to four-week-old maize plants were purchased from a local shop and transplanted to 0.5L pots filled with sandy loam soil. The soil, which consisted of 52% sand, 44% silt, and 4% clay, was sieved with a meshed sieve shaker (pore diameter=1.18mm). The plants were hydrated at an interval of 2 d and grown in an environmental growth chamber under a long-day condition (16h/8h light/dark cycle) with a photon flux density of 500 μmol m^−2^ s^−1^ for one week before soil drying. Light exposure was adjusted to a 3:3:6 ratio of 430:470:660nm using light-emitting diode lamps (PARUS, Korea). The temperature was maintained at 27±1 °C under a relative humidity (RH) of 70±5%.

### Xylem embolism induced by soil drying

Xylem embolism was induced by dehydration in 4–5-week-old maize plants. A total of 29 maize plants were well hydrated to field capacity, and initial mass (*w*_i_) was measured at the beginning of each experiment with a resolution of 0.1g. All these plants were 0.4–0.6 m tall and had 5–7 green leaves. The plants were grouped into six according to their dehydration period: the control group (*n=*3) was fully hydrated, and the five experimental groups were dehydrated for 2 d (*n=*4), 3 d (*n=*5), 4 d (*n=*6), 5 d (*n=*7), and 6 d (*n=*4). The test plants were exposed to water stress induced by soil drying for the given period in an environmental growth chamber and then weighed (*w*) just before X-ray imaging. At the end of experiments, planting pots were oven dried at >80 °C and then weighed (*w*_d_). The relative soil water content (RSWC) of each plant sample was evaluated using the following equation: RSWC=(*w* − *w*_d_) / (*w*_i_ − *w*_d_). The variation of soil water potential (Ψ_soil_) according to the different soil water content was also measured with a water potential sensor (MPS-6, Decagon Devices, Inc., USA) . The appearance of plants experiencing different soil drying periods and the corresponding RSWCs and Ψ_soil_ are reported in the Results section.

### Synchrotron X-ray micro-imaging experimental setup and procedure

Synchrotron X-ray micro-imaging experiments were conducted using the 6C Beamline at the Pohang Accelerator Laboratory (PAL, Pohang, Korea). An X-ray beam was generated using a 2.0 T superconducting bend magnet multipole wiggler (MPW14) operated at a critical energy of 12.08 keV. Three graphite filters 1mm thick were installed at the front end of the undulator beamline to absorb the long wavelength portion of the synchrotron radiation beam. Preliminary tests using 11–30 keV X-ray beams were performed to check whether X-ray exposure caused damage to plant leaves. Based on the preliminary tests, the energy of the monochromatic X-ray beam was fixed at 14 keV with an X-ray photon flux density of approximately 10^11^ photons s^−1^ mm^−2^ on a 300 mA run. The beam flux density of the 6C Beamline at the PAL was similar to that used in other X-ray imaging experiments on live plants (Brodersen *et al.*, 2010; Brodersen *et al.*, 2011; Brodersen *et al.*, 2013a; Brodersen *et al.*, 2013b; Choat *et al.*, 2015). Additionally, the sample-to-detector distance was set at 15cm, in which absorption- and phase-contrast effects were optimized. Each test sample was mounted on a traversing stage for the horizontal and vertical translations of the sample. The X-ray beam was filtered with a 0.5 mm-thick Si wafer to diminish strong light flux. The transmitted X-rays were then passed through a YAG:Ce scintillator crystal with a thickness of 50 μm; this scintillator converted X-rays to visible light, which was captured using a CCD camera (Andor Zyla, Ireland). An objective lens with a magnification of 10×(UPLASPO, NA 0.40, USA) was attached to the front of the camera. X-ray images were consecutively captured with a field of view (FOV) of approximately 1.7×1.4mm in the physical dimension. Spatial resolution was approximately 0.65 µm per pixel. The exposure time ranged from 100 to 500ms, depending on experimental conditions.

Plant samples were transferred from the growth chamber to the synchrotron X-ray imaging facility. A leaf from each intact maize plant was scanned and the status of the xylem vessels (embolized or not) was examined within ~2–6h after transferring the plant samples. Conditions inside the X-ray experiment hutch were different from the environmental conditions of the growth chamber, with a temperature of 25–27 °C, RH of 18–31%, and the irradiation condition adjusted to <3 μmol m^−2^ s^−1^. A maize leaf was taken from each intact plant, and a circular area, 25mm in diameter approximately, 12cm away from the leaf tip along the axial and lateral directions, was scanned. All scans for each maize plant including sample stage movements were completed within 1h. A mechanical shutter and attenuating plates were installed in front of the test sample to prevent unnecessary exposure to high-intensity X-ray radiation; thus, the test sample was exposed to the X-ray beam for only ~2min during the scans. X-ray exposure did not cause any noticeable effect on leaf tissues. Bright and dark field images were also recorded before and after the scanning experiment for each test sample. In post-image processing, a flat-field correction method was adopted to eliminate dark current noise from the CCD camera and the heterogeneity of the X-ray beam profile.

### Evaluation of embolism in xylem vessels

The inner morphological structure of maize leaves was observed from captured X-ray images. Gas-filled xylem vessels were clearly distinguished from water-filled xylem vessels and surrounding tissues. Water-filled vessels and surrounding tissues appeared as dark pixels, whereas gas-filled vessels appeared as bright pixels in grey-scale X-ray images (Supplementary Fig. S1). The inner diameter of xylem vessels, the length of water- and gas-filled vessels, and the spatial distribution of the two phases inside xylem vessels were analysed using ImageJ digital image processing software (Schneider *et al.*, 2012). The embolism ratio was defined as the number of gas-filled vessels to the number of total vessels within the scanned FOV region. Xylem vessels were classified into two groups: embolized and conducting vessels. The former included xylem vessels that were cavitated or partially filled with gas in the FOV (‘partially embolized’) and those filled with gas along the region of interest (‘fully embolized’); the latter included water-filled xylem vessels without gas bubbles in the FOV. The xylem vessels in each group were further divided according to vessel diameter in classes 2 μm wide. The numbers of conducting or embolized vessels were determined according to the vessel diameter by histogram analysis, and histogram values were fitted with a Gaussian distribution function.

## Results

### Drought stress induced in intact maize plants by soil drying

**Fig. 1.**
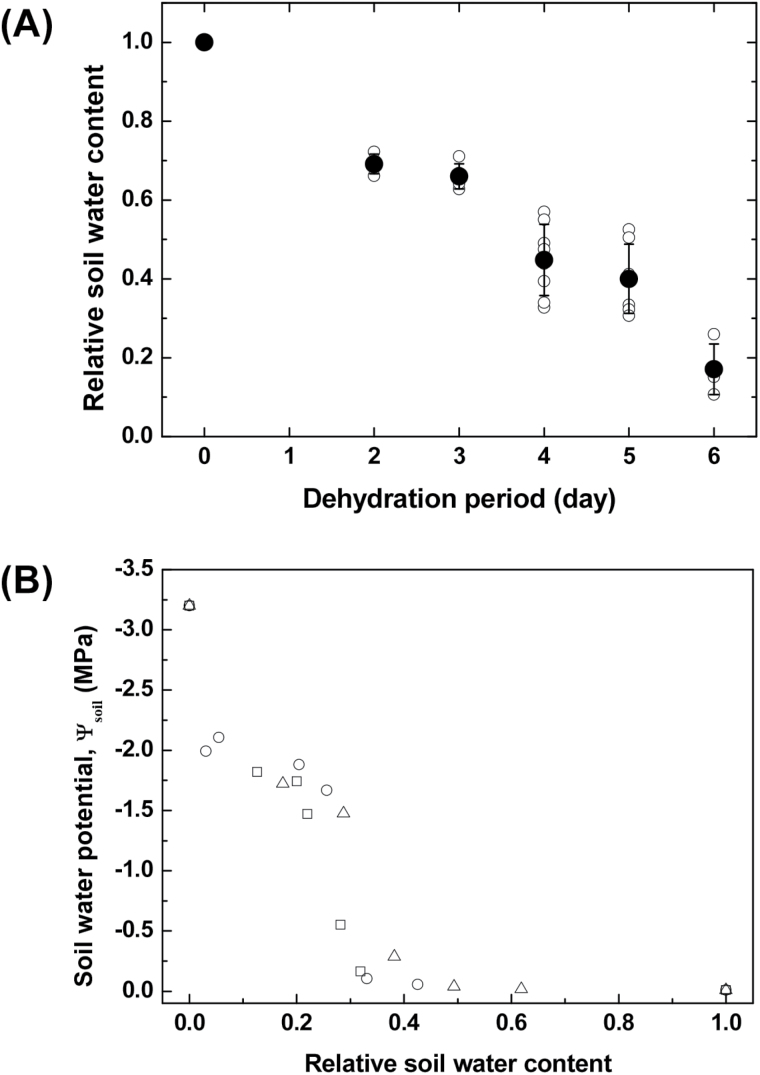
(**A**) RSWC of six different irrigation regimes for different soil-drying periods. Open symbols indicate experimental data of test samples (*n=*3, 4, 5, 6, 7, and 4 sample plants for 0, 2, 3, 4, 5, and 6 d of dehydration). Closed symbols represent statistically averaged RSWCs. Error bars indicate SD. (**B**) Variation of soil water potential (Ψ_soil_) according to RSWC. Open circle, square, and triangle symbols denote soil water potentials measured with changing RSWC in three repeated experiments (*n=*3).

The soil water content of the maize plant pots differed according to soil-drying period (Fig. 1A). We set the RSWC at 1.00 for the control (fully hydrated) group. For the two groups dehydrated for 2 and 3 d, RSWCs were maintained at 0.69 and 0.66, respectively. For the two groups dehydrated for 4 and 5 d, average RSWCs were 0.45 and 0.44, respectively. Over the course of the soil-drying period, RSWC decreased and maize leaves gradually withered but remained green until 4–5 d of dehydration. In the group dehydrated for 6 d, a few leaves severely wilted and turned yellow, and the average RSWC decreased to 0.17.

Fig. 1B shows the variation of Ψ_soil_ according to RSWC. When RSWC was >0.4, Ψ_soil_ was maintained at nearly the maximum value. When water content decreased to 0.2, Ψ_soil_ showed a steep decrease. For smaller RSWC between 0.2 and 0.05, the Ψ_soil_ slowly decreased to −1.8MPa. When RSWC approached zero, there was an exponential drop in Ψ_soil_. At this level of dehydration, maize leaves experienced severe water stress, turned yellow, and showed signs of withering.

### X-ray imaging of xylem embolism in intact maize plants

**Fig. 2.**
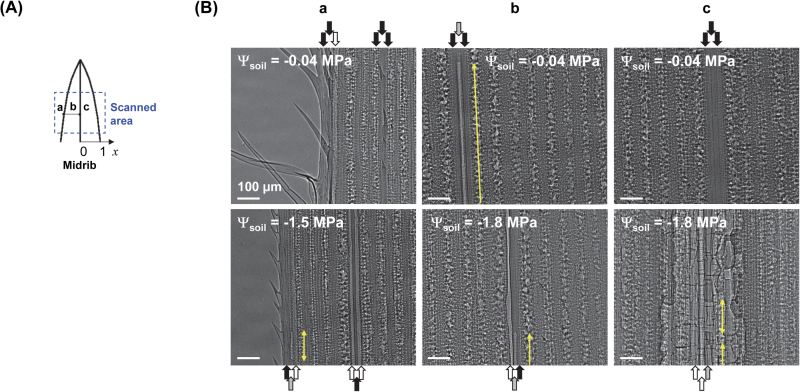
Direct *in vivo* visualization of the spatial distribution of xylem embolisms induced by soil drying in intact maize leaves. (**A**) Schematic of scanned area on a maize leaf. (**B**) Typical X-ray images showing the patterns of xylem embolism in intact maize leaves according to soil water potential (Ψ_soil_). The first row shows X-ray images for Ψ_soil_ of −0.04MPa. The second row corresponds to Ψ_soil_ of −1.5MPa in *a* and −1.8MPa in *b* and *c*, respectively. Columns *a*, *b*, and *c* were individually selected at different relative distances to the midrib (x): x=0, 0.5, and 1, respectively. Xylem vessels embolized entirely in the scanned FOV on maize leaves are called fully embolized vessels, and xylem vessels apparently cavitated or partially filled with gas in the FOV are called partially embolized vessels. The fully embolized vessels, partially embolized vessels, and water-filled vessels are represented by white, grey, and black arrows, respectively. Yellow arrows denote elongated gas bubbles in partially embolized vessels. Each X-ray image was captured from different leaves to show spatial embolism patterns from the midrib under different Ψ_soil_ conditions. This figure is available in colour at *JXB* online.

The vulnerability to drought of vascular plants was assessed by scanning vascular bundles in maize leaves in a specific FOV (Fig. 2A) by high-resolution synchrotron X-ray imaging. Vascular bundles of maize leaves are arranged in a series, and these bundles included one protoxylem vessel between two large metaxylem vessels. These typical xylem vascular bundle arrangements could be clearly observed in two-dimensional X-ray radiograms (Fig. 2B). Although there are often many narrow, water-conducting vessels and tracheids surrounding these vessels in a vascular bundle, they were not clearly distinguishable on X-ray images. X-ray images also provided other structural characteristics of xylem vessels, such as xylem diameters and qualitative information about the contrast between water and air. Helical thickenings of protoxylem vessels appeared as dark lines in the images.

Captured X-ray images showed that overall embolism in xylem vessels gradually increased with Ψ_soil_ (Fig. 2B). When Ψ_soil_ was −0.04MPa, most embolisms were observed in one metaxylem vessel of the xylem bundles in the marginal region of maize leaves (relative distances to midrib *x=*1). Embolism sometimes occurred in one xylem vessel of each xylem bundle located at 0 < *x* < 1 and the vessel was sometimes partially embolized (indicated by grey arrows on Fig. 2B). We found that embolism rarely occurred at the midrib (*x=*0). Severe drought stress led to leaf wilting. At a Ψ_soil_ of −1.5MPa, numerous vessels were embolized in the xylem vessels: embolism was commonly observed in the midrib region and occurred in protoxylem and metaxylem vessels in one xylem bundle. Representative X-ray images of intact maize leaves revealed that overall embolism increased with decreasing Ψ_soil_.

**Fig. 3.**
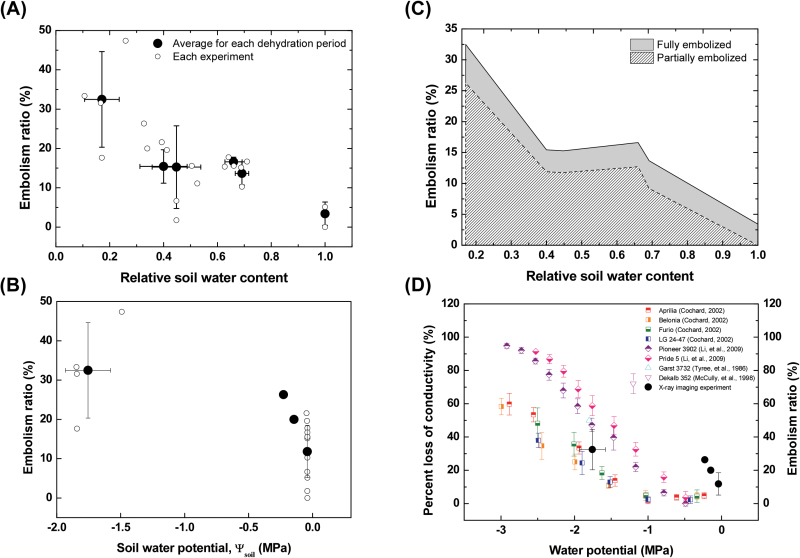
Xylem embolism ratios in maize leaves under different soil water contents. (**A**) Variation of xylem embolism incidence rate of intact maize leaves according to RSWC and (**B**) embolism ratio in intact maize leaves according to Ψ_soil_. Small open circles represent experimental data (*n=*21), and large closed circles denote the averaged values of the samples dehydrated for 0, 2, 3, 4, 5, and 6 d. Horizontal and vertical error bars indicate SDs. (**C**) Variation of embolized status in xylem vessels according to RSWC. The portion of fully embolized vessels is indicated by the grey-shaded area, and that of partially embolized vessels is depicted by the hatched area. (**D**) Comparison of directly measured loss of conductivity in maize leaves with other previous results of indirect and invasive measurements (Tyree *et al.*, 1986; McCully *et al.*, 1998; Cochard, 2002; Li *et al.*, 2009). Red, orange, green, and blue squares indicate the PLC in four maize genotypes of Aprilia, Belonia, Furio, and LG 24–47, respectively (Cochard, 2002). Purple and pink diamonds indicate PLC in two maize genotypes of Pioneer 3902 and Pride 5, respectively (Li *et al.*, 2009). Cyan triangle indicates the embolism ratio observed at the internode of maize, Garst 3732 (Tyree *et al.*, 1986). Magenta reversed triangle indicates the embolism ratio observed in late metaxylem vessels in the roots of maize, Dekalb 352 (McCully *et al.*, 1998). Closed circle symbols represent the statistically averaged embolism ratios obtained in this study. This figure is available in colour at *JXB* online.

Subsequently, average embolism ratios in the scanned FOV of intact maize leaves were quantitatively assessed (Fig. 3A). In the control group, the ratio of embolized vessels to total vessels was 3.4%, which indicated the basal level of embolism before exposure to dehydration. At a RSWC >0.25, 10–15% of vessels were embolized. When the RSWC decreased to <0.25, an embolism ratio of approximately 30% was induced. By comparison with baseline embolism levels, our data indicate that the water stress caused by d 2–6 of dehydration resulted in embolisms in intact maize leaves. Fig. 3B shows the embolism ratio in intact maize leaves with respect to Ψ_soil_. When Ψ_soil_ was maintained at ~0MPa, approximately 10% of plant samples experienced embolism. However, when Ψ_soil_ decreased as low as −1.8MPa, the embolism ratio increased on average to 32%.

Interestingly, most of the cavitated xylem vessels were partly filled with gas in the FOV of intact maize leaves (Fig. 3C). The proportion of fully embolized vessels was nearly constant at ~5% regardless of the RSWC. The ratio of partially embolized vessels was also maintained at approximately 12% until a RSWC >0.4. This sudden increase in embolism occurrence under severe water stress, in which RSWC greatly decreased and some leaves wilted, caused the proportion of partially embolized vessels to subsequently increase without noticeable changes in the proportion of fully embolized vessels. Temporal variations in the number of total vascular bundles per leaf and the number of water-filled, fully embolized, or partially embolized xylem vessels are summarized in Table 1.

**Table 1.** Characteristics of the water contents of xylem vessels

Day	Number of vascular bundles	Number of water-filled xylem vessels	Number of fully embolized xylem vessels	Number of partially embolized xylem vessels	Embolism ratio (%)
2	15	38	1	6	15.6
	11	28	2	3	15.2
	13	35	2	2	10.3
3	13	33	1	5	15.4
	15	37	2	6	17.8
	14	35	2	5	16.7
4	15	42	0	3	6.7
	19	42	4	11	26.3
	15	36	3	6	20.0
	19	56	0	1	1.8
	17	40	2	9	21.6
5	17	41	2	8	19.6
	15	40	1	4	11.1
	15	38	2	5	15.6
6	19	39	7	11	31.6
	19	38	3	16	33.3
	19	30	2	25	47.4
	17	42	2	7	17.6

In Fig. 3D, embolism formation in maize leaves measured by X-ray micro-imaging in this study was compared to the vulnerability to embolism formation reported in previous studies for maize leaves, internodes, and roots (Tyree *et al.*, 1986; McCully *et al.*, 1998; Cochard, 2002; Li *et al.*, 2009). All of the previous results were obtained from indirect measurements of the loss of hydraulic conductivity [per cent loss of conductivity (PLC)] due to embolism, and invasive measurements such as acoustic emission and cryo-SEM techniques. In the present study, embolism ratios were quantitatively derived from direct visualization of water and gas phases inside xylem vessels of intact maize leaves. When Ψ_soil_ remained within 0 to −0.5MPa, embolism ratios were quite high compared to those from indirect measurements. However, considering the redundancy of xylem vessels, the PLC in this case could be estimated to be lower than the embolism ratio. When Ψ_soil_ decreased to −1.8MPa, the embolism ratios were comparable to PLC from indirect measurements.

**Fig. 4.**
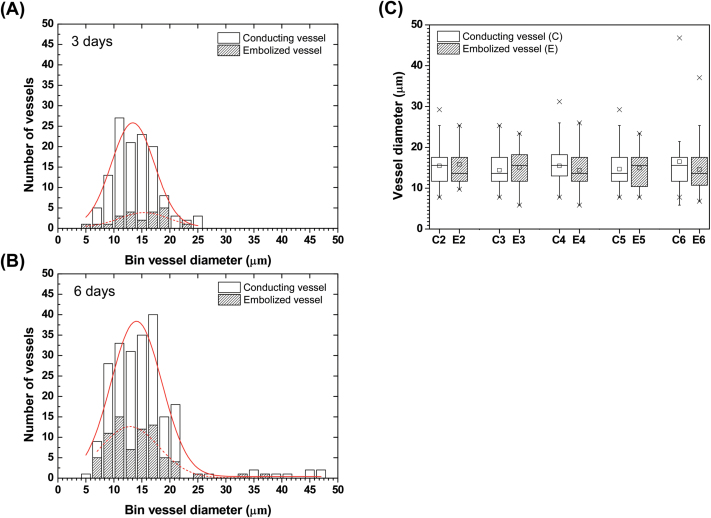
Xylem vessel size and embolism occurrence. (**A**, **B**) Histograms of conducting (white bars) and embolized xylem vessels (hatched bars) of plant samples dehydrated for 3 d and 6 d, respectively. For soil dried for 3 d, 126 xylem vessels in three intact maize plants were analysed, whereas for soil dried for 6 d, 222 xylem vessels in four plants were investigated. (**C**) Comparison of vessel diameters of conducting (white box) and embolized xylems (hatched box) of samples (*n=*3, 3, 5, 3, and 4) dehydrated for 2, 3, 4, 5, and 6 d, respectively. The small rectangle represents the mean value, and crosses denote 1st and 99th percentiles. This figure is available in colour at *JXB* online.

Vessel diameter distributions of conducting and embolized vessels in the FOV of sample plants were compared after 3 and 6 d of dehydration (Fig. 4A and B). The peak diameter of Gaussian distributions for conducting xylem vessels in the group dehydrated for 3 d (*n=*3) was 13 μm, whereas that for embolized vessels was 16 μm. For the group dehydrated for 6 d (*n=*4), peak diameter of embolized vessels was 13 μm, which was smaller than that of conducting vessels, 14 μm. Peak diameters of conducting and embolized vessels were extracted from histograms of the groups dehydrated for 2, 3, 4, 5, and 6 d, and results were compared and are shown in Fig. 4C. For the groups dehydrated for 3 and 5 d, the peak diameters of embolized vessels were greater than those of conducting vessels. For the groups dehydrated for 2, 4, and 6 d, the peak diameters of embolized vessels were smaller than those of conducting vessels. These inconsistent peak diameters in histogram distributions imply that xylem diameter is not strongly correlated with embolism occurrence in maize leaves.

**Fig. 5.**
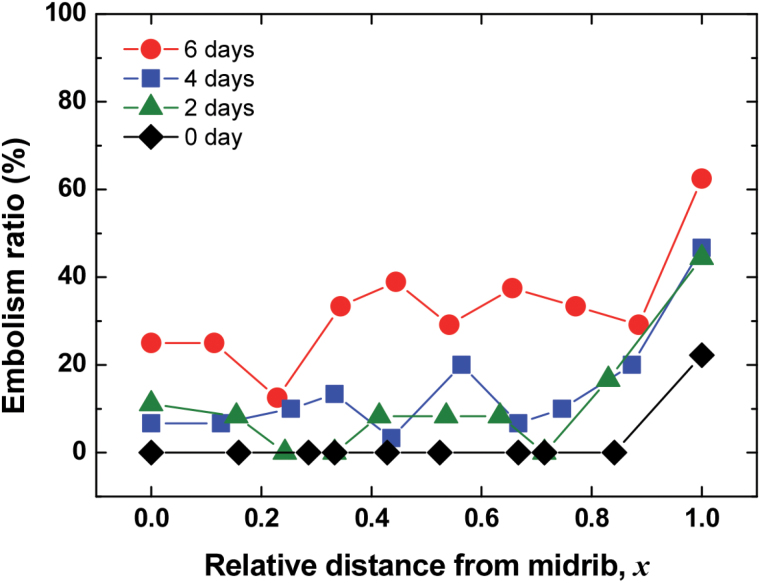
Spatial variations in embolism ratios with respect to relative distance from the midrib. Red circles denote embolism ratios of plant samples dehydrated for 6 d (*n=*4); blue rectangles, green triangles, and black diamonds denote embolism ratios of samples dehydrated for 4 d (*n=*5), 2 d (*n=*3), and 0 d (*n=*3), respectively. Each symbol represents statistically averaged embolism ratio in xylem vessels. This figure is available in colour at *JXB* online.

To understand the effect of morphological structures in xylem vessels on embolism occurrence, we analysed the spatial distribution of xylem embolism under water stress from 0–6 d of dehydration. Water content in intact xylem vessels was directly observed, and results showed that embolism occurred more easily in xylem vessels positioned at the edge of leaves (*x=*1) than in other vessels, regardless of the degree of water stress (Fig. 5). More than 40% of xylem vessels at *x=*1 were embolized under moderate water stress, and severe water stress induced embolism in approximately 60% of xylem vessels in the marginal region. By contrast, other vessels positioned within 0 ≤ *x* < 1 experienced ≤20% embolism under moderate water stress and even under severe water stress from 6 d of soil drying, and only ≤40% of xylem vessels were embolized. In general, vessel diameters of midribs were greater than those of other xylem vessels. However, embolism occurrence exhibited no distinguishable features, except for a greater occurrence in xylem vessels located farthest from the midrib.

**Fig. 6.**
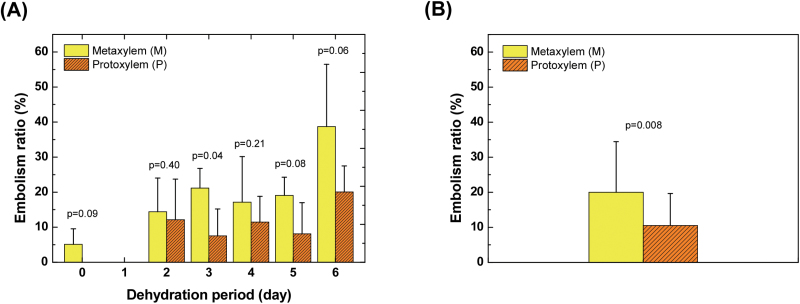
Difference in embolism occurrence ratios in metaxylem (M) and protoxylem (P) vessels. (**A**) Specific xylem-type embolism occurrence according to dehydration periods. Each bar represents the ratio of gas-filled specific-type vessels (M or P) to the total number of corresponding vessels (*n=*3, 3, 3, 5, 3, and 4 for 0, 2, 3, 4, 5, and 6 d of dehydration). Yellow bars denote embolism ratios of metaxylem vessels, and orange hatched bars indicate embolism ratios of protoxylem vessels. (**B**) Comparison of embolism ratios in metaxylem and protoxylem vessels with respect to xylem vulnerability (*n=*21). Unpaired one-tailed t-test results indicate high and moderate significance. Error bars indicate SD. This figure is available in colour at *JXB* online.

Embolisms occurred more frequently in metaxylem vessels than in protoxylem vessels (Fig. 6). Statistically, there was sometimes an even distribution of embolism between the protoxylem and metaxylem vessels under moderate water stress (2 and 4 d of dehydration). However, native embolisms were observed only in metaxylem vessels. In addition, there were approximately twice as many metaxylem vessels embolized as protoxylem vessels on d 3 and 5 of dehydration, and even under severe water stress on 6 d of dehydration. Overall, our investigation of embolism ratios in 21 intact maize plants revealed that metaxylem vessels were more vulnerable to cavitation than protoxylem vessels (Fig. 6B).

## Discussion

Direct observation and detailed analysis of embolism occurrence with internal anatomical structures of xylem vessels demonstrated the strong potential of X-ray micro-imaging as a direct and non-destructive visualization tool for intact plants. X-ray micro-imaging was free from any methodological artefacts induced by the pre-treatment procedures of other conventional indirect measurements, such as acoustic emission and cryo-SEM. In these techniques, stems, leaves, and roots are usually excised, which could cause xylem vessels to embolize. In addition, the X-ray micro-imaging technique used in this study did not result in any adverse effects on leaf tissues of intact plants after exposure to the X-ray beam. Although the temperature in the region of the leaf exposed to the X-ray may have slightly increased, X-ray exposure did not promote cavitation or embolism in xylem vessels. The xylem vessels were not cavitated or embolized during beam exposure within the scanned FOV of fully hydrated maize leaves, except for native embolisms (Figs 2 and 5). In addition, it did not cause any noticeable morphological changes or visible damage to the plant samples tested in this study. These results support that X-ray micro-imaging can provide reliable quantitative data for embolism occurrence in individual xylem vessels.

For the first time, direct observation of xylem embolism on intact maize plants has been possible, using a high-resolution synchrotron X-ray imaging technique that revealed low embolism occurrence after water stress. We used directly observed X-ray images to evaluate the level of xylem embolism induced by soil drying for 2–6 d. The level of native embolism remained at approximately 3–4%, and the level of xylem embolism ranged from 10% to 15% when the RSWC was >0.4. Up to a maximum of ~30% xylem embolism was observed, even after plant leaves started wilting (Fig. 3A and B). These results should be carefully compared with the level of xylem embolism hydraulically estimated in previous studies (Fig. 3D). Because the interconnectivity of xylem vessels may enable sap flow to bypass the embolized xylem vessels, the embolism ratio represents a plausible maximum loss of hydraulic conductivity. Water fluxes in the conducted and embolized xylem vessels also have to be taken into consideration when comparing our results with the results of previous studies. Nevertheless, the embolism ratios estimated in this study based on X-ray images are similar to results obtained in a previous study showing that highly stressed maize leaves (i.e. water potential <−1.5MPa) had low vulnerability to embolism (Cochard, 2002). The lower level of xylem embolism in leaves explains why maize can survive under severe drought.

The low ratio of embolized to functional xylem vessels in maize leaves suggests that cavitation is not routine on a daily basis, contrary to previous reports (Tyree *et al.*, 1986; McCully *et al.*, 1998; Li *et al.*, 2009) (Fig. 3D). Previous ultrasonic acoustic measurements (Tyree *et al.*, 1986) revealed that 50% of vessels are embolized at internodes of maize plants under drought. In another study using cryo-SEM (McCully *et al.*, 1998), 70% of xylem vessels in roots of well-watered maize plants were observed to be embolized at midday. These results suggested embolism and refilling on a daily basis in maize. However, the level of xylem embolism was relatively lower in our study than in previous works. The frequency of embolism occurrence observed by acoustic emission and cryo-SEM methods in those previous studies suggest that these techniques create artefacts that affect studies on the water content in xylem vessels.

Direct observation of xylem embolism in maize leaves also revealed that most of the embolized vessels were partially gas-filled under water stress (Table 1). In previous studies, partial embolism was detected during the water-refilling process in several embolized xylem vessels in petioles of *Helianthus* (Canny, 1997), and the roots of monocotyledonous (McCully *et al.*, 1998) and dicotyledonous crop plants (Buchard *et al.*, 1999). However, the partial embolism observed by cryo-SEM in those previous studies could be the result of artefacts. In our study, the partially gas-filled conduits appeared as large elongated gas bubbles in xylem vessels (Fig. 2B). The amounts of water and air in the partially embolized xylem vessels were approximately evaluated from their corresponding X-ray images. Unfortunately, it was nearly impossible to specify the exact locations of water in the partially gas-filled xylem vessels. Therefore, it was difficult to prove where the interrupted air bubbles were generated and to assert whether partial embolism occurred in the embolism or water-refilling process. To understand the implication of partially embolized xylem vessels, further in-depth studies are required with more advanced bio-imaging techniques that provide a wider FOV and higher temporal resolution.

Spatial distributions of xylem embolism induced by various soil-drying conditions were directly observed in intact maize leaves. Most previous studies have examined cavitation and embolism events in woody plants (Holbrook *et al.*, 2001; Nardini *et al.*, 2001; Brodersen *et al.*, 2013a; Brodersen *et al.*, 2013b; Totzke *et al.*, 2013; Zwieniecki *et al.*, 2013; Choat *et al.*, 2015; Cochard *et al.*, 2015). However, cavitation and embolism in monocotyledonous crop plants, especially maize, are rarely investigated. In intact maize leaves, xylem embolism only occurred in some of the vessels. Only one or two xylem vessels in a xylem bundle were generally embolized, except under extremely severe soil drying. These findings confirm that maize plants have structural xylem traits against embolism, as detailed below.

The first interesting point in the observed characteristic spatial distribution of embolism occurrence was that it was not affected by vessel diameter in the intact maize leaves under water stress (Fig. 4). Previous studies on woody plants (Cochard and Tyree, 1990; Sperry and Tyree, 1990; Hargrave *et al.*, 1994; LoGullo *et al.*, 1995; Davis *et al.*, 1999; Lintunen *et al.*, 2013) have reported that large vessels tend to cavitate more easily than small vessels. The present study showed apparently conflicting results. Although the xylem vessels at the midrib had the largest diameters, xylem embolism barely occurred in the midrib (Fig. 5). It may be that the no significant relationship was found in this study between vessel diameter and the degree of xylem embolism because there was a relatively small range of diameters in the xylem vessels in maize leaves, as shown in Fig. 4A and B.

The second interesting point was the observed spatial distribution of xylem embolism in maize leaves (Fig. 5). Irrespective of soil-drying conditions, distal ends of maize leaves were more easily embolized than xylem vessels in other locations. Even in fully hydrated plants, the only embolized vessels were found in the xylem vessels in the margin of maize leaves. The high embolism ratio in the most peripheral veins (*x=*1, Fig. 5) indicated that xylem vessels in the leaf margin were more vulnerable to cavitation and embolism. These different embolism occurrence patterns can be explained by different photosynthetic rates in plant leaves (Brodribb *et al.*, 2007; Chen *et al.*, 2008). If leaf margins have higher rates of photosynthesis, the consequent greater demand for water supply and evaporative loss makes the xylem vessels vulnerable. Water potential gradients within leaves may also result in higher levels of embolism formation in vascular bundles at the leaf periphery. Xylem vessels in the leaf margin are the most closely linked to hydathodes, which might enable air bubbles to enter more easily as compared to xylem vessels in the central region. We did not measure water potential in intact maize leaves; however, a previous study reported that steep gradients in water potentials can be established in transpiring leaves of *Tradescantia virginiana* L. (Shackel and Brinckmann, 1985). The different embolism patterns may also be related to xylem vessel connectivity. The marginal vascular bundles may be less connected to neighbouring bundles, while the central ones have higher connectivity. Embolism occurrence in xylem vessels at the midrib would deliver a fatal blow to stable water transport. Meanwhile, the proportion of embolized xylem vessels sequentially increased in the regions away from the midrib. Although curling in plant leaves is well known to be due to volume changes of bulliform cells (Mauseth, 1988), the spatial distribution of increasing xylem embolism from the midrib to the distal ends may also contribute to the curling and flexural bending of maize leaves (Tyree *et al.*, 2002; Sack and Scoffoni, 2013) and the gradual decrease in hydraulic conductance under intensified water stress.

The third interesting point was the different rates of embolism according to xylem vessel type. Overall, metaxylem vessels were more vulnerable to embolism than protoxylem vessels (Fig. 6). This difference was due to the varied anatomical structures of metaxylem and protoxylem vessels. Protoxylem vessels are known to be less lignified compared to metaxylem vessels. The highly lignified walls of metaxylem vessels increase the hydrophobicity of the surface of the xylem vessel wall, triggering cavitation and embolism (Donaldson, 2001; Sperry, 2003; Wheeler and Stroock, 2009). The number and biophysical properties of pits in the metaxylem vessels may influence the higher vulnerability to embolism in metaxylem vessels than in protoxylem vessels. Protoxylem vessels also seem to function as a safety system to maintain water transport even under intense water stress and as a facilitator of water refilling of adjacent embolized metaxylem vessels (Canny, 2001). Different structural traits of protoxylem and metaxylem vessels and their connectivity with other tissues are believed to be closely related to cavitation resistance and safe water transport in vascular plants.

In conclusion, direct observation of embolism occurrence induced by soil drying in intact maize leaves revealed the rates of embolism within a natural range of Ψ_soil_ and indicated characteristic structural strategies for decreasing vulnerability to embolism. The present results provide insights into drought-induced xylem embolism in monocotyledonous crop plants and help elucidate plant strategies for survival in response to drought stress.

## Supplementary data

Supplementary data are available at *JXB* online.

Fig. S1. Discrimination of air-filled and water-filled xylem vessels based on grey values.

Supplementary Data
