# Supplementary material for: Direct observation of local xylem embolisms induced by soil drying in intact Zea mays leaves
Source: J Exp Bot. 2016 Mar 5;67(9):2617–26. doi: 10.1093/jxb/erw087 (PMC4861012; doi:10.1093/jxb/erw087)
Supplement: Supplementary Data [file supp_67_9_2617__index.html]

Direct observation of local xylem embolisms induced by soil drying in intact Zea mays leaves — Direct observation of local xylem embolisms induced by soil drying in intact Zea mays leaves — Supplementary Data 

# Direct observation of local xylem embolisms induced by soil drying in intact *Zea mays* leaves

## Supplementary Data

Data files

- Supplementary\_Figure\_1.pdf - Supplementary Data
