# Supplementary material for: Direct observation of local xylem embolisms induced by soil drying in intact Zea mays leaves
Source: J Exp Bot. 2016 Mar 5;67(9):2617–26. doi: 10.1093/jxb/erw087 (PMC4861012; doi:10.1093/jxb/erw087)

Direct observation of local xylem embolisms induced by soil drying in intact maize leaves

Jeongeun Ryu, Bae Geun Hwang, Yangmin X Kim, and Sang Joon Lee Supplementary Information

Figure S1. Discrimination of air-filled and water-filled xylem vessels based on gray values

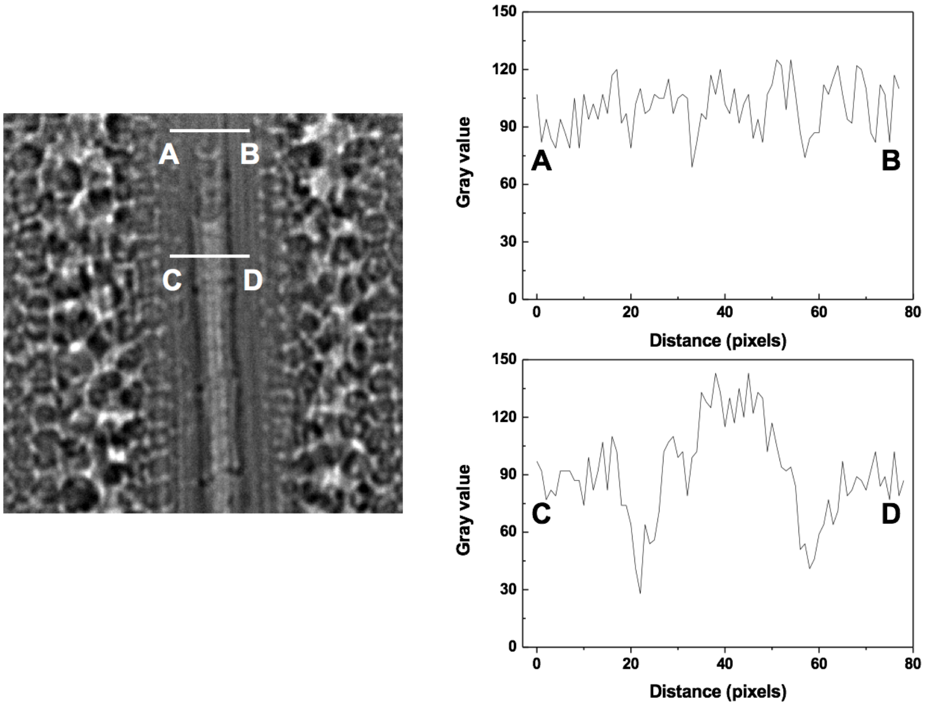

Supplement: Supplementary Data [file supp_erw087_Supplementary_Figure_1.pdf]
